# A clue to clarify the origin of Omicron

**DOI:** 10.1098/rsos.250926

**Published:** 2025-10-22

**Authors:** Fernando Martinez-Hernandez, Claudia Irais Muñoz-Garcia, Emilio Rendon-Franco, José Antonio Ocampo Cervantes, Rigoberto Hernandez-Castro, Angelica Olivo-Diaz, Mirza Romero-Valdovinos, Pablo Maravilla, Nelly Raquel Gonzalez-Arenas, Guiehdani Villalobos

**Affiliations:** ^1^ Hospital General Dr Manuel Gea Gonzalez, Ciudad de Mexico, Mexico; ^2^ Departamento de Produccion Agricola y Animal, Universidad Autonoma Metropolitana—Unidad Xochimilco, Coyoacán, Ciudad de México, Mexico; ^3^ Centro de investigaciones biológicas y acuícolas de Cuemanco, Unidad Xochimilco, Universidad Autonoma Metropolitana, Ciudad de México, Mexico

**Keywords:** SARS-CoV-2, COVID-19, Omicron, *Mus musculus*, *Rattus norvegicus*

## Abstract

This study utilized faecal samples from synanthropic rodents collected during September–December of 2020 from a public park in southern Mexico City, previously identified as positive for SARS-CoV-2, to perform molecular identification of the viral variant. Typing was successful for two *Mus musculus* and one *Rattus norvegicus*, revealing Omicron variants BA.5.2 (from a rat) and BA.5.1.17 (from mice). Additionally, confirmation from a reference centre and Global Initiative on Sharing All Influenza Data (GISAID) analysis verified the presence of the Omicron variant. By considering three primary hypotheses regarding Omicron's origin and integrating these findings with concurrent events in Mexico during the initial wave of SARS-CoV-2, it is reasonable to suggest that the variant’s emergence may stem from a combination of a ‘silent spread’ (indicating circulation among populations with limited viral monitoring and sequencing) and a ‘mouse origin’ (where the progenitor of Omicron transitioned from humans to mice, acquiring mutations that enhanced its ability to infect that host, before re-emerging in humans). Consequently, our results indicate that Omicron was present in urban rodents a year prior to its official emergence, amplified by the conditions existing during the first wave of COVID-19 in Mexico.

## 1. Background

After the pandemic SARS-CoV-2 virus began to spread in November 2019, surveillance of variants was initiated. This led to the establishment of classifications such as variants under monitoring (VUMs), variants of interest (VOIs) and variants of concern (VOCs) [1–3].

One of the features of a VOC is that it is likely to have a higher transmissibility, virulence, a change in disease presentation or a decreased sensitivity to diagnostic tests, treatments, vaccines or other countermeasures [3,4]. Notable VOCs include Alpha (B.1.1.7), Beta (B.1.351), Gamma (P.1), Delta (B.1.617.2) and Omicron (B.1.1.529) [5–7]. Omicron was first identified in South Africa in November 2021 and was quickly classified as the fifth VOC [5,6]. Since its identification, it has rapidly disseminated globally and became the predominant lineage.

The entry of SARS-CoV-2 into its host cell is considered to be mediated by the interaction of its spike (S) protein with the host cell receptor, angiotensin-converting enzyme 2 (ACE2) [8]. Most of the variations in the S protein emergence with the presence of mutations in receptor-binding domain (RBD) are described as VOCs [1–3].

The 77 full-genome Omicron sequences had around 55 (range 48–92; mean 54.6 ± 5.2) mutations relative to the original Wuhan Hu-1 sequence (mostly non-synonymous mutations), based on initial genomic analyses. While this number of mutations is not much higher than for the Delta VOC (average 34.4 ± 2.6), the pattern of mutations is worrying [3], with approximately 30 of these lying in the viral Spike gene (all non-synonymous mutations) and 15 of these are located within the RBD site, hence the importance of this site [9], a major target of infection and immune response [3].

Subsequent analyses have identified more mutations in the Spike and other proteins of Omicron, as well as five separate sublineages (BA. 1, BA. 2, BA. 3, BA. 4, BA. 5), as well as sublineages within BA. 1 and BA. 2 [2,5,7].

The origins of Omicron are unknown; proposals for its origins are still being debated. Currently, three major hypotheses have been proposed: (i) Omicron might have been spread ‘silently’ rather than circulating persistently in patients in populations with inadequate viral monitoring and sequencing [10,11]; (ii) it may have emerged in one or a few individuals with chronic infection, allowing an environment for optimal long-term viral adaptation prior to identification in a population that is actively monitored [10,11]; (iii) given the mutation patterns observed *in vitro* for both mouse and human environments, the ancestral virus of the Omicron variant transitioned from humans to mice quickly collecting the mutations needed to infect mice and then back to humans [11,12].

In the beginning, S-proteins of SARS-CoV-2 isolates during early pandemic did not bind to mouse ACE2 [8,13–15]. Nevertheless, Omicron and its sublineages with an accumulation of multiple amino acid substitutions in RBD such as K417N, N440K, G446S, S477S, E484K, Q493R, Q498R and N501Y were shown to be capable of infecting mice [13].

During September–December 2020, while the first wave of COVID-19 cases were reported in Mexico City, a study focused on the identification of intestinal pathogens in synanthropic rodents captured in a public park, reported that seven rodents, four *Mus musculus* (Linnaeus, 1769) and three *Rattus norvegicus* (Berkenhou, 1769) were tested positive for SARS-CoV-2 with cycle threshold (Ct) values greater than 30 by one-step quantitative reverse transcription polymerase chain reaction (qRT-PCR), using the SARS-CoV-2 (Open Reading Frame, ORF1ab; and Nucleocapsid, N genes) real-time PCR detection kit (Viasure, CerTest Biotec, Zaragoza, Spain). Thus, this study molecularly evidenced the presence of SARS-CoV-2 in rodents in Mexico, but its lineage was not identified [16]. The objective of this study is to determine the variant of SARS-CoV-2 found in the samples from these positive rodents.

## 2. Methods

### 2.1. Sources and descriptions of samples

The samples analysed in this work came from a previous survey on SARS-CoV-2 detection in urban free-ranging rats [16]. Briefly, the rodents were captured with commercial galvanized wire mesh box traps (Tomahawk Live Trap-like), measuring 30 × 20 × 14 cm, baited with oats, vanilla essence, peanut butter and corn tortilla, anaesthetized with chloroform and euthanized by cervical dislocation. The specimens were transferred to the laboratory for morphological identification, age classification (using weight and body length) and determination of reproductive status.

### 2.2. Spike gene amplification

RNA purification from the gut contents of the rodents, previously analysed and positive to SARS-CoV-2 diagnosis [16], was performed on an automated platform (Maelstrom 9600, TANBead, Taipei, Taiwan) using a commercially available extraction protocol (TANBead Viral Nucleic Acid Extraction Kit, Tiangen, Beijing, China). The complementary DNA (cDNA) was synthesized by reverse transcription reaction using a random primer with 9 μl of total RNA and the reverse transcriptase (SuperScript II (Invitrogen, CA, USA) according to the manufacturer’s instructions.

**Figure 1.**
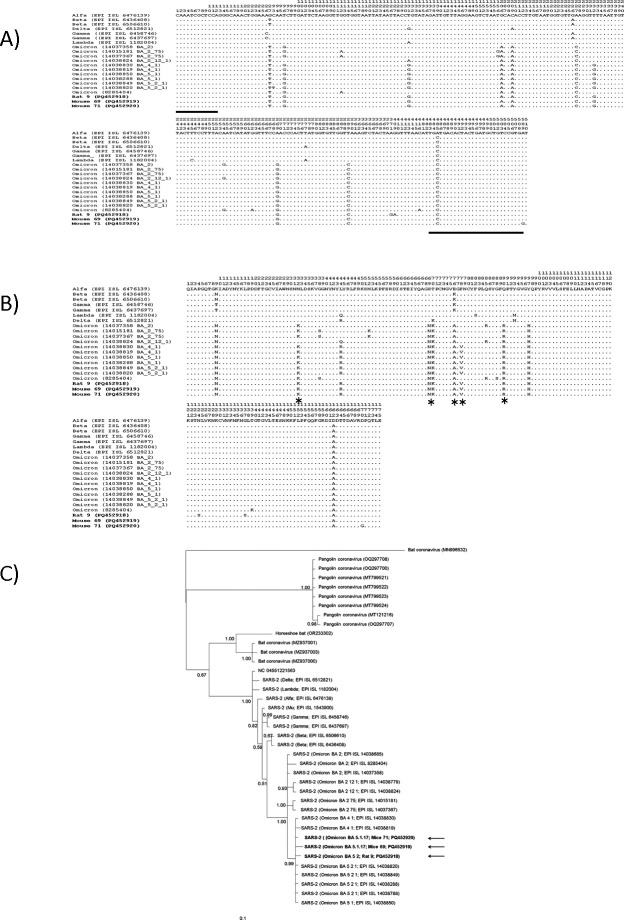
Multiple alignment and Bayesian phylogenetic tree based on Spike partial sequences of SARS-CoV-2 variant obtained in the GenBank and GISAID database. (A) Multiple alignment of nucleotides; (B) Multiple alignment of amino acids and (C) Bayesian phylogenetic tree. The asterisks show characteristic amino acid in Omicron variant, the black arrows and bold letters indicate the sequences obtained in the present study and the black lines indicate the position of the internal primers.

Amplification of Spike gene was performed by nested PCR using the set of specific primers for SARS-CoV-2 Spike, custom-designed based on the available sequences of different SARS-CoV-2 variants, from the Global Initiative on Sharing All Influenza Data (GISAID) database [17] (EPI_ISL_6476139 and EPI_ISL_2666072 for Alpha variant, EPI_ISL_6506610 and EPI_ISL_6436408 for the Beta variant, EPI_ISL_6437697 and EPI_ISL_6458746 for the Gamma variant, EPI_ISL_6512821 for Delta, EPI_ISL_1182004 for Lambda, EPI_ISL_1543900 for Mu, and EPI_ISL_6842167 and EPI_ISL_11798493 for Omicron). Both pairs of primers hybridize with common regions in all the above-mentioned SARS-CoV-2, while the amplified internal region contains specific nucleotides for each variant, thus the sequence obtained can approximate the type of variant present in the samples. The external primer set was spikeFext 5´-CTT CTA ACC AGG TTG CTG TTC-3´ and spikeRext 5´-GCT TGG AAC AGG AAG AGA ATC-3´, which amplifies a 777 bp region, and nested primers spikeF1 5´-AAG TCA GAC AAA TCG CTC CA-3´ and spikeR2 5´-GAT GCT GTC CGT GAT CCA C-3´ to amplify a 521 bp fragment between nucleotide positions 1216 and 1737 of the Spike gene (reference sequence: NC_045512.2). At the protein level, this region comprises amino acids 408–583, a total of 175 amino acids that contain a fragment of the RBD site and a fragment of the SD1 site harbouring 14 of 15 mutations characteristic of Omicron (figure 1A). An amount of 25 μl total volume mixtures were first prepared for the first amplification reaction under the following conditions: 1× PCR buffer (100 mM Tris-HCl, pH 8.3, 500 mM KCl) 3, 1.5 mM MgCl_2_, 10.5 pM of each primer set, 200 mM dNTP mix, 0.01 mg albumin, 1 U of *Taq polymerase* (Invitrogen, CA, USA), 5 μl cDNA. The PCR was performed with a Veriti thermal cycler (Applied Biosystems, CA, USA) under the following conditions: 5 min at 94°C and 35 cycles of denaturation at 94°C for 1 min, annealing at 58°C for 1 min, and extension at 72°C for 1 min, and then a final extension at 72°C for 7 min. For the nested amplification, reactions similar to the 25 μl with the final concentrations already mentioned but the external primer pairs were performed with the following PCR programme: initial 5 min denaturation at 94°C, 35 cycles at 94°C for 50 s, 50 s at 62°C and 50 s at 72°C and a final extension of 7 min at 72°C.

### 2.3. Sequencing and phylogenetic analysis

The amplicons of the SARS-CoV-2 Spike gene fragments were purified using QIAquick PCR purification Kit (Qiagen, Germany) and sequenced by the Sanger method using internal primers in both directions. The sequences were analysed using the MEGA software, generating consensus sequences that were submitted to the GenBank to obtain the access numbers PQ452918-PQ452920. The consensus sequences were queried on the GISAID web portal [17] at the AudacityInstant search tool to determine the most similar genomes in the EpiCov database in terms of lineage, variant, clade, location and collection date among related genomes. Phylogenetic analysis was performed with Mr. Bayes 3.2 software using the Bayesian inference. This analysis included at least one member of each SARS-CoV-2 genetic variant identified to date as obtained from the GISAID database and outgroups, including sequences in GenBank, as well as related coronaviruses (bat = MN996532, OR233302, MZ937000-1 and MZ937003; pangolin = OQ297708, OQ297700, MT121216, MT799521-24 and OQ297707). Additionally, median-joining network analysis was constructed using NETWORK 4.611. Two phylogenetic constructions were created, one based on the year of sampling and another based on the country of sample collection. Haplotype networks were established under default settings and assumptions.

Furthermore, RNA samples were sent to a reference centre in Mexico (Institute of Genomic Medicine—INMEGEN, Ministry of Health, Mexico) for verification of the viral variant, here SARS-CoV-2 variants were routinely identified through massive sequencing with Artic v. 4.1 primers.

## 3. Results

Although amplicons of the partial sequence of *spike* were generated in all but seven of the positive rodent samples, only two mouse samples and one rat sample passed quality and purity thresholds for Sanger sequencing. These sequences were then analysed and submitted to GenBank (PQ452918-20).

**Figure 2.**
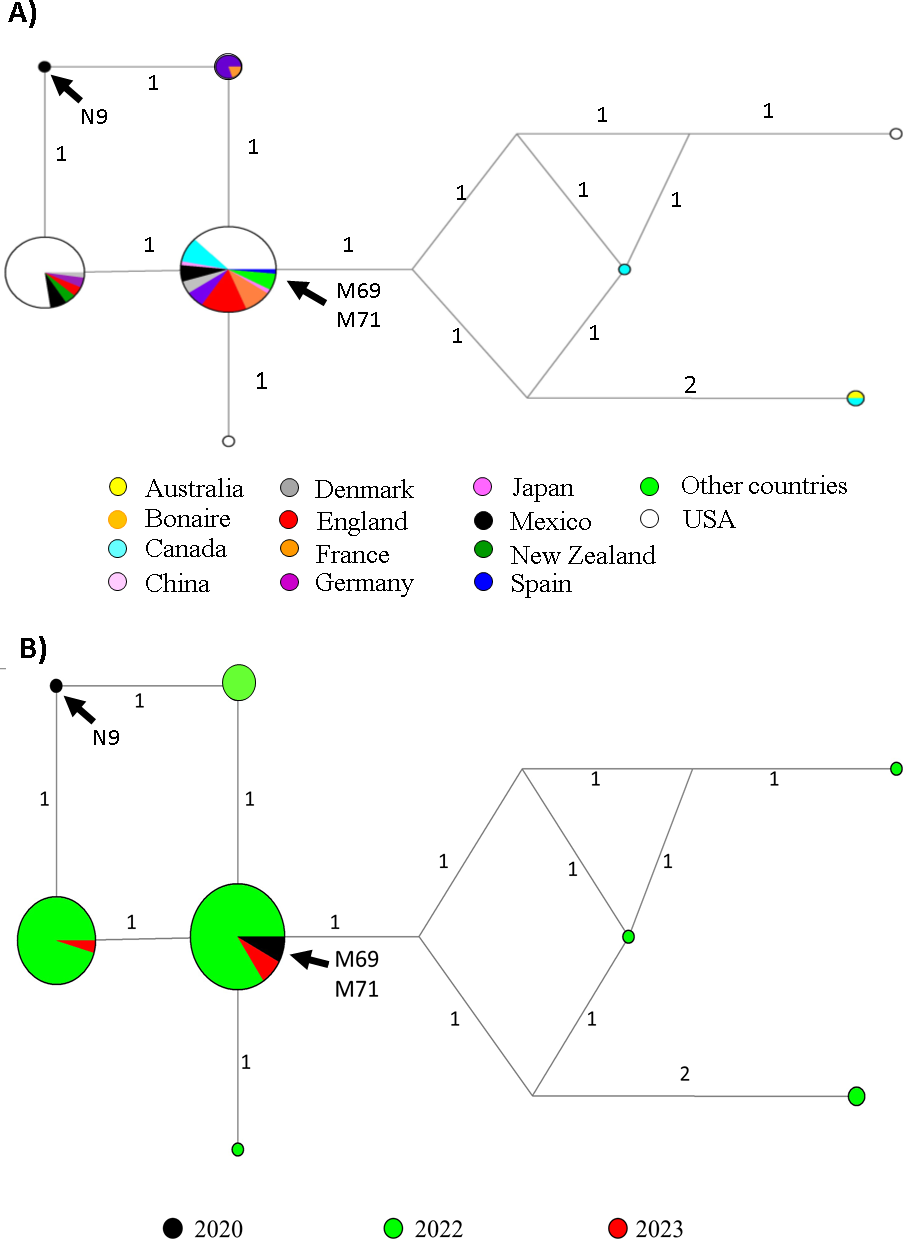
Haplotype network trees illustrating the geographic distribution of the Omicron variant sequences (A) and the timeline of sample collection for Omicron sequences (B). The Mexican samples identified in this study are highlighted in black; N9 corresponds to the *Rattus norvegicus* sample, while M69 and M71 correspond to *Mus musculus*. The numbers on branches in the haplotype network indicate mutational changes; the circle sizes reflect haplotype frequencies.

Sequence comparisons with AudacityInstant from GISAID determined these sequences to be suggestive of the Omicron variant BA.5.2 (from a rat) and BA.5.1.17 (from mice), notably, the sequenced fragments contained the N440K, S477N, E484A, Q498R and F486V, mutations specific to this variant and the last specific for the BA.4 and BA.5 sublineages [18] (figure 1A,B). Bayesian inference also showed that the three sequences fell within the Omicron clade, specifically to the subclade that contains variants BA.4 and BA.5 (figure 1C). The haplotype trees showed that the haplotypes of mice samples were distributed in a central scattered area, suggesting that the samples from mice were located to that reported samples from human patients in various countries (Figure 2A), which were mainly reported in the year 2022, with a handful rejected in 2023 (figure 2B). By contrast, the haplotype from the rat reference sample was located in the peripheral clustering area, and there were no matched haplotypes in either GenBank or GISAID.

Additionally, results from the reference centre in Mexico corroborated the presence of Omicron variants (from mice 69) with a sequencing coverage of 60.69%.

## 4. Discussion

Although there’s been concern about synanthropic urban rodents since COVID-19 was first reported, epidemiologists suggest that only a small proportion of *R. norvegicus’s* population is infected by SARS-CoV-2 (up to 6%, as revealed by qRT-PCR analysis) [16,19–22]. One study of 79 *R*. *norvegicus* trapped in New York City (NYC) in fall 2021 identified four qRT-PCR-positive rats with sequences matching to lineage B, which had been predominant in NYC during the early pandemic in spring 2020 [19]. Combined with the need for immediate new vaccination, therapeutic and prevention strategies at the time of the pandemic, these observations may have obscured the surveillance focus on synanthropic rodents as hosts and/or carriers of SARS-CoV-2 and its variants. Reports about the origin of Omicron have emphasized phylogenetic analyses and figures showing the mutations of the S-protein on a phylogenic tree with respect to its divergence from the Wuhan Hu-1 sequence and the fact that there are many sequences with different patterns of mutations between possible closest ancestors of Omicron, which could date from approximately mid-2020 [6,11]. Our finding in rodents on the Omicron variant, which ha support from phylogenetic analyses, further supports the model that rodents are reservoirs, despite not having been the dominant variant in Mexico during the fourth wave as of late 2022. Of course, the possibility of contamination between samples could be considered; however, this bias is unlikely due to the care taken during sample handling, sourcing and processing of samples.

As has been argued for other pathogens, when a population structure exhibits a high recombination rate among all members, the structure transforms into a network rather than a tree. However, occasionally a highly successful individual emerges whose frequency increases rapidly, producing an epidemic outbreak [23]. However, the geographical and temporal differences between the Omicron variant detected in rodents in Mexico and the first outbreak of the same variant in South Africa, almost a year later, may be due to the fact that they were independent evolutionary events, with synanthropic rodents (*M. musculus* and *R. norvegicus*) being the common hosts that promoted the detonation of this variant.

Controversial strategies during the first COVID-19 wave in 2020 by the Mexican Secretary of Health with a high number of deaths, mainly of medical staff and insufficient diagnostic tests, are further documented [24–26]. Variant B.1 was the only variant reported by any of the 202 sequences from Mexico on GISAID from 10 January to 31 December 2020. with variant B.1.1.222 being predominant. The United States, for example, transmitted 28 059 sequences during the same period, and by October 2020 more than 126 000 genomes had been submitted worldwide to GISAID, including more than 2200 from Latin America and the Caribbean [27].

In light of the three most relevant hypotheses for the Omicron origin mentioned above, and under the assumption of our findings, the coincidence of these events in the timeline of Mexico City in the first SARS-CoV-2 wave would lead to venture a hypothetical origin of this VOC stemming from the synthesis of the ‘silent spread’ and ‘mouse origin’ hypotheses. The detection of Omicron-like viruses in a potential urban rodent host 1 year before its official detection suggests that Omicron may have circulated under the radar due to insufficient new variant monitoring and surveillance.

## Data Availability

The obtained sequences in this research were submitted to GenBank (PQ452918, PQ452919 and PQ452920).
